# Effects of multiple N, P, and K fertilizer combinations on strawberry growth and the microbial community

**DOI:** 10.1371/journal.pone.0293088

**Published:** 2023-11-27

**Authors:** Xue Bai, Kaili Liu, Tiao Ning, Changjun Deng, Li Wang, Diyan Li, Tao Wang, Jing Li

**Affiliations:** 1 Engineering Research Center for Urban Modern Agriculture of Higher Education in Yunnan Province, School of Agriculture and Life Sciences, Kunming University, Kunming, China; 2 Antibiotics Research and Re-evaluation Key Laboratory of Sichuan Province, Sichuan Industrial Institute of Antibiotics, School of Pharmacy, Chengdu University, Chengdu, China; 3 Yunnan Hanzhe Technology Co., Ltd., Kunming, China; 4 Xinhui Forestry Science Institute, Jiangmen, China; 5 Shangri-La Zangmei Agricultural Technology Co., Ltd., Diqing, China; 6 Shangri-La City Small Berry Research and Development Center, Diqing, China; Hainan University, CHINA

## Abstract

Nitrogen (N), phosphorus (P), and potassium (K) exert various effects on strawberry (*Fragaria ananassa Duchesne*) yields. In this study, we employed an orthogonal experimental design (T1-T9) with three fertilization treatments (N, P, and K) at three levels to identify an optimal fertilization scheme for strawberry cultivation. The effects of fertilizer combinations the rhizosphere soil microbial community were also explored by using bacterial full-length 16S rRNA and fungal ITS (internal transcribed spacer) sequencing (30 samples for each analysis). The results showed that the average plant height and leaf area of the fertilized groups were 24.6% and 41.6% higher than those of the non-fertilized group (T0). After 60 d of planting, the sucrase activity in the T6 group increased by 76.67% compared to the T0 group, with phosphate fertilizer exerting a more significant impact on sucrase activity. The T6 treatment group had the highest alpha diversity index among bacterial and fungal microorganisms, and had a different microbial community structure compared with the control group. The most abundant bacterial taxa in the strawberry rhizosphere soil were Proteobacteria, Bacteroidota, and Acidobacteriota, and the most abundant fungal phyla were Monoblepharomycota, Glomeromycota, and Mucoromycota. Application of the optimal combined fertilizer treatment (T6) significantly increased the abundance of Proteobacteria and altered the abundance of *Gemmatimonas* compared to other treatment groups. Notably, *Gemmatimonas* abundance positively correlated with strawberry plant height and soil N, P, and K levels. These findings indicated that the relative abundance of beneficial bacteria could be enhanced by the application of an optimal fertilizer ratio, ultimately improving strawberry agronomic traits.

## Introduction

The influence of mineral fertilizer and water on crop yield is of paramount importance in agricultural systems [1–3]. However, overfertilization may lead to soil and water pollution and pose a serious threat to food safety [4,5]. Therefore, it is increasingly necessary to manage fertilizer well in Chinese agriculture. Strawberry (*Fragaria ananassa Duchesne*) is a perennial herb. Due to its fruit fragrance and sweet and sour taste, it is highly suitable for consumption. The fruit is rich in a variety of active ingredients that are beneficial to human health, including amino acids, vitamins, calcium, magnesium, iron, and other minerals. It has high nutritional and medicinal value and is popular with the public. Known as the "Queen of fruits," it is widely cultivated all over the world. [6]. Strawberry is a shallow-rooted crop with high nutrient demand and a strong ability to absorb fertilizer. However, strawberry roots are very sensitive to fertilizer in the soil environment, and excessive or insufficient fertilizer application has a significant impact on fruit quality and plant nutrient absorptio [7,8]. China has a large strawberry planting area and ranks first in the total output of strawberries in the world, second only to the United States and Mexico among 79 countries [9]. Therefore, strawberry cultivation in China requires a large amount of fertilizer.

Nitrogen, phosphorus, and potassium are the main mineral fertilizers. Nitrogen is the most essential nutrient in crop production as it plays a crucial role in cell division [10,11], and nitrogen deficiency reduces crop yield and quality [12,13]. Phosphorus nutrients are necessary for photosynthesis and have been shown to increase wheat yields [14–16], and potassium is also essential [17]. Potassium (K), the second most abundant element found in plant tissues following nitrogen (N) [18,19], exerts a significant influence on critical physiological processes. Its pivotal role encompasses the improvement of water absorption efficiency and the enhancement of fruit quality [20].

Although a large number of studies have shown that a reasonable combined application of nitrogen, phosphorus, and potassium fertilizer can improve crop performance, yield, and quality [21–23], most studies only focus on the impact of fertilization on the crop surface [24–26]. There have been few reports on the agronomic characteristics and rhizosphere soil characteristics of strawberries when combined with N, P, and K (NPK) fertilizer. Therefore, based on a scientific and reasonable experimental design, this study investigates the effects of the combined application of N, P, and K fertilizer on strawberry growth and rhizosphere soil characteristics. Traditional detection methods and advanced microbial molecular technology were employed to conduct a comprehensive analysis. This research aims to deeply explore the impact of N, P, and K fertilizer on strawberry agronomic traits and rhizosphere soil characteristics. The objective of this study is to enhance the theoretical foundation regarding the impact of combined nitrogen, phosphorus, and potassium fertilizer application on strawberry production. Additionally, it aims to provide guidance for scientific fertilization practices in strawberry cultivation, facilitating the maintenance of high yields, superior fruit quality, and ecological balance within the soil.

## Materials and methods

### Study site and experimental design

Three fertilizer components at three concentrations each were assessed in this study. Component A was nitrogen fertilizer, component B was phosphorus fertilizer, and component C was potassium fertilizer. The application levels of each component were as follows: component N, 90, 225, and 360 kg/hm^2^; component P_2_O_5_, 68, 124, and 180 kg/hm^2^; and component K_2_O, 260, 360, and 460 kg/hm^2^. Group T0 comprised unfertilized samples (Table 1). There were three biological replicate plots for each treatment group. A total of 30 experimental plots were randomly arranged. All fertilizers were basally applied at a single time point and fully mixed with the soil. Each set of three adjacent plots received the same treatment. Strawberry plants were randomly selected from the front, middle, and back of each plot for observation and recording of agronomic indexes at 30, 60, 90, and 120 d. The relevant indices (described below) were measured at 30, 60, 90, and 120 d after transplantation. To ensure normal growth, regular unified field management strategies were used.

**Table 1:** L9 orthogonal test design.

treatments	factor		
	N(kg/hm^2^)	P(kg/hm^2^)	K(kg/hm^2^)
**T1**	90	68	260
**T2**	90	124	360
**T3**	90	180	460
**T4**	225	68	360
**T5**	225	124	460
**T6**	225	180	260
**T7**	360	68	460
**T8**	360	124	260
**T9**	360	180	360

The test site was in the greenhouse next to the College of Agronomy and Life Science, Kunming University, Yunnan Province. The site had flat terrain, soil that had not previously been planted with strawberries, a deep soil layer, and good drainage. The basic soil nutrient indices were as follows: organic matter content, 32.12 g/kg; total nitrogen content, 2.18 g/kg; alkali-hydrolyzed nitrogen content, 94.65 mg/kg; total phosphorus content, 1.34 g/kg; available phosphorus content, 93.58 mg/kg; total potassium content, 7.74 g/kg; available potassium content, 253.88 mg/kg; pH, 5.6.

The strawberry type tested was the early-maturing powder fruit variety ‘Fanyu’, which has extremely vigorous growth potential, a high fruit-setting rate, low requirements for climatic conditions, a low deformity rate, and a high yield. Urea (N ≥ 46.3%) was used as the N fertilizer. Phosphorus fertilizer chooses calcium superphosphate with P_2_O_5_≥16.0%. The potassium fertilizer selected was potassium sulfate (K_2_O ≥ 52.0%) for agricultural use.

### Plant indices

#### Determination of agronomic characteristics

Part of the agronomic traits of strawberries were measured 30, 60, 90, and 120 d after planting. Plant height is measured from the highest point at the center of the strawberry plant to the root of the plant. Measure the leaf length, leaf width, petiole length and petiole diameter based on the central leaf of the third flattened functional compound leaf with the heart leaf outward. The leaf area was calculated as leaf length × leaf width × 0.73 [27].

#### Rhizosphere soil characteristics

In each of the 10 treatment groups, 6 strawberry plants were randomly selected [28] from the front and middle of one biological replicate plot, the middle and back of another biological replicate plot, and the front and back of the last biological replicate plot. The soil was collected from these plants with the "shaking root method". Soil was first removed from the root surface (representing the 0–5 cm soil layer) after the plants were pulled from the soil. The rhizosphere soil (5–20 cm soil layer) was then sampled with specialized soil sample collection tools. Large rocks, residual roots, and branches were removed. For each treatment group, rhizosphere soil collected from each of the three plots was thoroughly mixed and evenly divided into thirds for analyses of soil enzyme activity, soil nutrients, and microorganisms. After soil samples were collected, mixed, and divided, they were labeled, sealed in sterile plastic bags, placed on ice, and returned to the laboratory. One portion of each sample was then air-dried, and the other portion was frozen at -80°C prior to further use.

Urease, sucrase, and catalase activities were measured in the rhizosphere soil samples. The total nitrogen, total phosphorus, total potassium, and organic matter contents were all measured, as was the pH of each sample. The rhizosphere soil microbial communities (i.e., bacterial and fungal community diversity) were assessed with high-throughput sequencing at 120 d after transplantation as described below.

### Statistical analysis software

Statistically significant differences between the mean values of each treatment group were assessed with least significant difference (LSD) tests in SPSS 20.0. Differences were considered significant at *p* < 0.05.

### DNA extraction and microbial 16S rRNA gene sequencing and analysis

Total genomic DNA was extracted from soil samples using the TGuide S96 Magnetic Soil/Stool DNA Kit (Tiangen Biotech (Beijing) Co., Ltd.) following the manufacturer’s instructions. The quality and quantity of the extracted DNA were assessed with 1.8% agarose gel electrophoresis. The DNA concentration and purity were quantified on a NanoDrop 2000 UV-Vis spectrophotometer (Thermo Scientific, Wilmington, Delaware, USA). Full-length 16S rRNA genes were amplified with the 27F (5’- AGRGTTTGATYNTGGCTCAG-3’)/1492R (5’-TASGGHTACCTTGTTASGACTT-3’) [29] primers. Fungal internal transcribed spacer (ITS) regions were amplified with the ITS1F (5’-CTTGGTCATTTAGAGGAAGTAA-3’)/ITS4 (5’-TCCTCCGCTTATTGATATGC-3’) [30] primer pair. PCR was performed with the KOD One PCR Master Mix (TOYOBOLife Science) using the following thermocycling conditions: initial denaturation, 95°C for 2 m-in; 25 cycles of denaturation at 98°C for 10 s, annealing at 55°C for 30 s, and extension at 72°C for 1 min 30 s; and a final extension at 72°C for 2 min. The resulting amplicons were quantified, then normalized equimolar concentrations of the amplicons were pooled and sequenced on the PacBio Sequel II platform (Beijing Biomarker Technologies Co., Ltd., Beijing, China).

Sequences were classified into operational taxonomic units (OTUs) using the standard 97% similarity threshold in USEARCH (version 10.0). Taxonomy annotation was performed on the ASVs with the Naive Bayes classifier in QIIME2 [31] using the SILVA database [32] (release 138.1) with a confidence threshold of 70%. Alpha community diversity was assessed in QIIME2. Beta diversity calculations were conducted with principal coordinate analysis (PCoA). Differences in bacterial abundance and diversity were determined with one-way analysis of variance (ANOVA). Linear discriminant analysis (LDA) coupled with effect size (LEfSe [33]) analyses were applied to evaluate the differentially abundant taxa. The online platform BMKCloud (https://www.biocloud.net) was used to analyze the sequencing data.

## Results

### Impacts of combined N, P, and K fertilizer application on strawberry phenotypes

To explore the effects of combined N, P, and K fertilizer application on strawberry phenotypes. We used nine groups of fertilizer application throughout the strawberry growth period. As a result, fertilizer application showed a significant increase in plant height compared to the unfertilized control T0. The T6 treatment group produced the tallest plants of 27.76 cm at 120 d. However, there were no statistically significant differences in plant height between fertilizer treatment groups. The strawberry leaf area clearly increased throughout the 120 d growth period in fertilized plants. However, the growth rate of the leaf area slowed after 90 d, indicating that strawberry leaf growth was relatively stable beyond that point. Notably, at 60 days, plants in group T7 demonstrated significantly higher leaf area compared to other groups, reaching a maximum of 49.86 cm^2^. The petiole length was highest in the T6 and T9 groups at 30 d, exhibiting an 84.51% increase compared to the T0 treatment.

**Fig 1:**
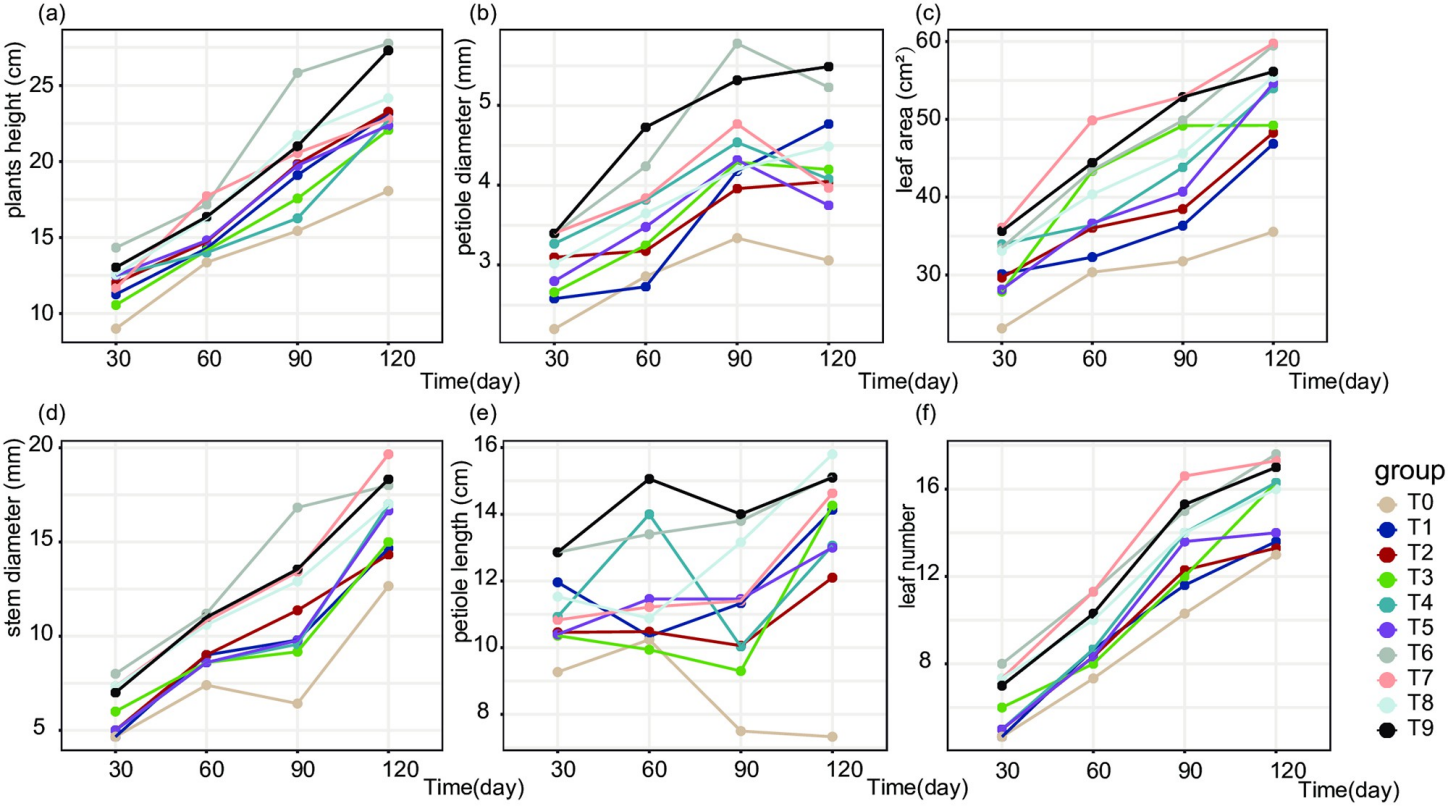
Effects of different nitrogen, phosphorus, and potassium fertilization treatments on strawberry phenotypes from 30–120 d after planting.

During the entire growth phase, the T9 group had the greatest increase in petiole diameter, followed by the T6 group. Compared to the T0, the T9 treatment showed increases of 54.55%, 65.38%, 59.28%, and 79.41% at four time points, respectively. At the initial three stages, stem diameter was highest in the T6 group, with the maximum value occurring at 30 d after planting. The T7 and T8 groups also showed large increases in stem diameter (71.67% and 57.30%, respectively) compared to the T0 treatment. However, there were no significant differences observed between the fertilized treatment groups. The number of strawberry leaves gradually increased over time after fertilizer application. By 120 d, the number of strawberry leaves reached a maximum; the T6 group had the largest number of leaves, followed in descending order by groups T7, T9, T3, T4, T8, T1, T2, and T0 (Fig 1).

### Effects of combined N, P, and K fertilizer treatment on enzymatic activities in rhizosphere soil

**Fig 2:**
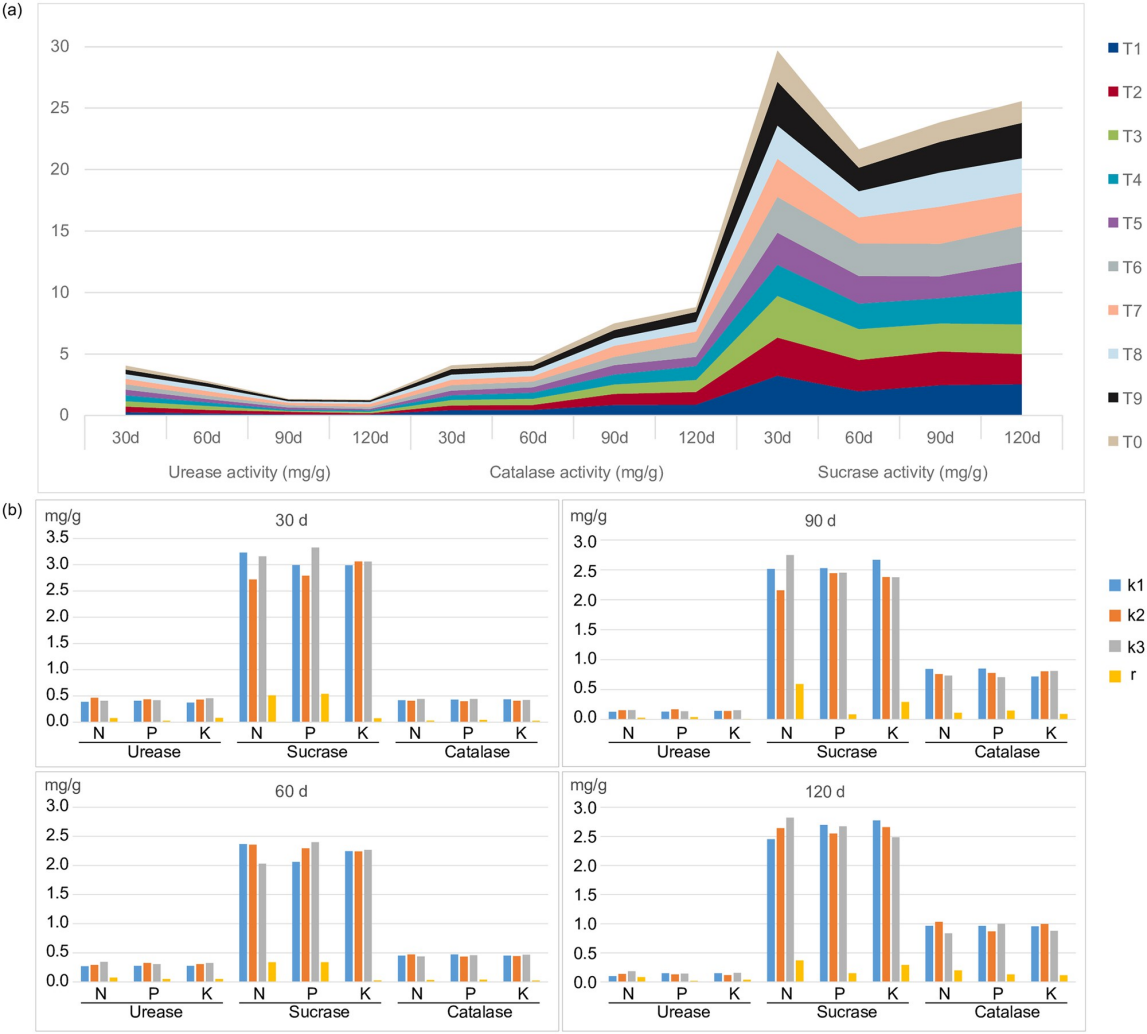
**(a)** Urease, sucrase, and catalase activity in strawberry rhizosphere soil. **(b)** Range analysis and level difference analysis of urea, sucrose, and catalase activities in strawberry rhizosphere soil with combined nitrogen, phosphorus and potassium fertilizer application within each column, k1, k2, and k3 represent levels one (low), two (medium), and three (high) for each factor. r represents the range of factors between levels.

The activities of urease, sucrase, and catalase in the strawberry rhizosphere were shown to be enhanced by the application of nitrogen, phosphorus, or potassium fertilizer. Specifically, urease activity in the rhizosphere soil surrounding strawberries was significantly increased in the T7 treatment group. However, urease activity consistently decreased throughout the strawberry development process (Fig 2a). Nitrogen treatment had the largest impact on urease activity, followed by potassium treatment, then phosphorus (Fig 2b).

At 60 d after planting, sucrase activity was significantly higher in treatment groups T6, T3, and T2 compared to T0, with increases of 76.67%, 67.33%, and 69.33%, respectively (Fig 2a). The order of fertilizer components influencing sucrase activity was P > N > K, with factor A corresponding to the highest sucrase activity in the rhizosphere soil under consistent water conditions (Fig 2b). Catalase activity in the strawberry rhizosphere soil progressively increased from 30 to 120 d after stabilizing. At 120 d, treatment group T6 showed the largest increase in catalase activity, followed by the T2 and T4 groups. Although no significant differences were observed between the remaining treatment groups, all the groups treated with fertilizer had higher catalase activity than the unfertilized control (T0) (Fig 2a).

### Effects of combined N, P, and K fertilizer on rhizosphere soil composition

**Fig 3:**
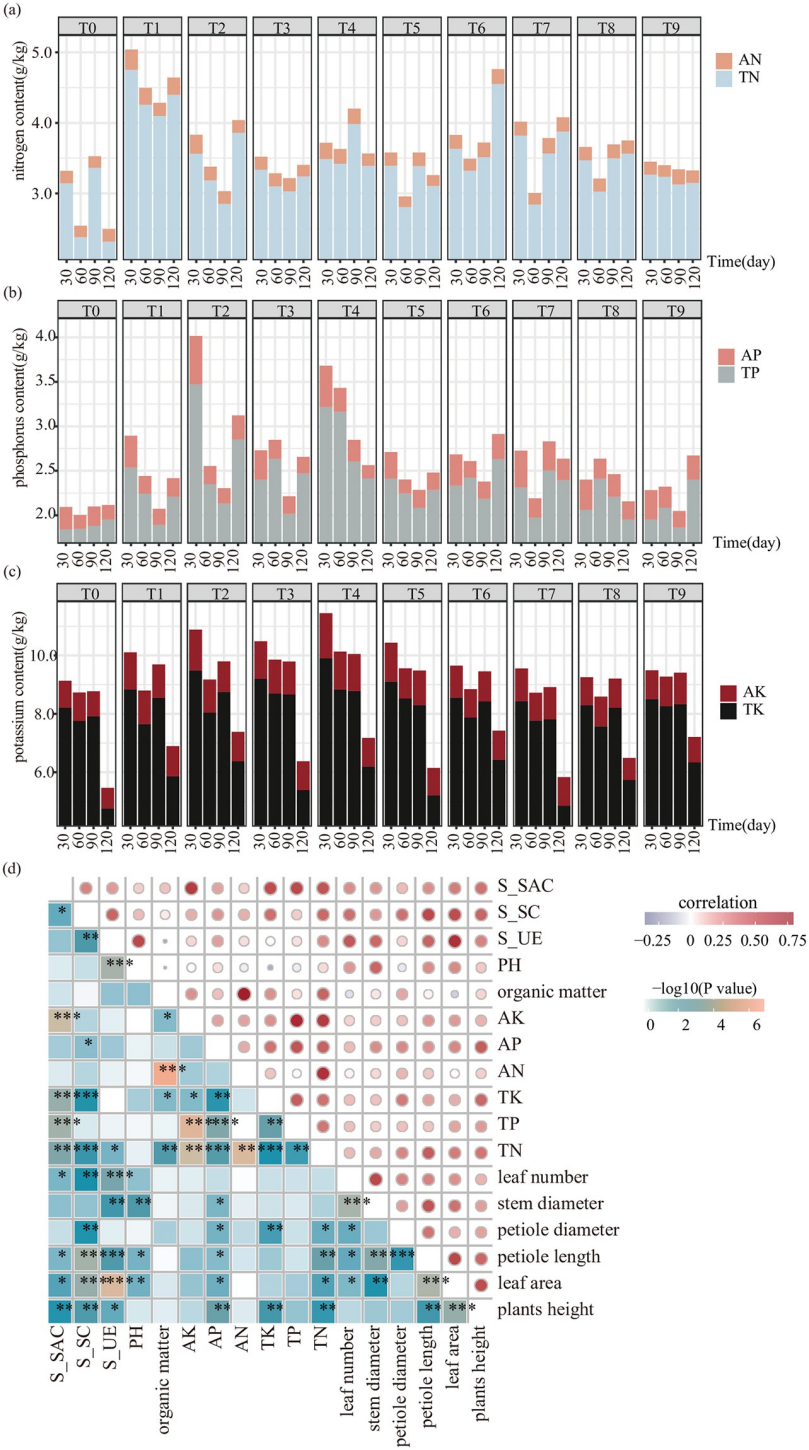
**(a)** Nitrogen content in the rhizosphere soil after fertilizer treatment. AN, total nitrogen; TN, available nitrogen. (**b)** Phosphorus content in the rhizosphere soil after fertilizer treatment. AP, total phosphorus; TP, available phosphorus. **(c)** Potassium content in the rhizosphere soil after fertilizer treatment. AK, total potassium; TK, available potassium. (**d)** Correlation matrix for strawberry phenotypes and soil composition characteristics. Blue and red dots represent negative and positive correlations, respectively. *p < 0.05, **p < 0.01, ***p < 0.001.

The lack of fertilizer in the soil is an important factor limiting crop growth, so this study examined the NPK content in the soil. The result showed that the total nitrogen content was higher in the strawberry rhizosphere soil at all time points after the combined application of nitrogen, phosphorus, and potassium fertilizer compared to the unfertilized group (Fig 3a–3c). Overall, the total nitrogen content fluctuated. Compared with T0, the total nitrogen content was significantly increased under the T1 treatment, and changes in the alkali-hydrolyzed nitrogen content followed a similar pattern as the total nitrogen content. This observation may be attributed to the varying nitrogen requirements of strawberries at different growth stages. The T2 and T4 groups had significantly higher total phosphorus content than the other treatment groups. Specifically, at 90 d, the total phosphorus content in the strawberry rhizosphere soil increased by 38.30% in the T4 treatment group compared to the T0 group. The available potassium content in the rhizosphere was highest during the first 30 d for each group, after which it gradually decreased to a stable level. The total potassium content in the strawberry rhizosphere soil was significantly lower at 120 d compared to the first three time points. T2 and T4 showed significant improvements, with little overall change observed in the available potassium content. After fertilizer application, the available potassium content was higher in the rhizosphere soil of treated samples than in the T0 group, with the most significant increase observed in the T1 treatment group. To further explore the relationships between strawberry phenotypes and levels of nitrogen, phosphorus, and potassium in the soil, a correlation analysis was conducted for plant phenotypes and soil composition (Fig 3d). Plant phenotypic traits showed positive correlations with the total phosphorus and available nitrogen contents in the soil and were significantly positively correlated with soil enzyme activity *(p* < 0.05) (Fig 3d).

### Effects of N, K, and P fertilizer on the rhizosphere soil microbial community

We next sought to gain a comprehensive understanding of the effects of combined nitrogen, phosphorus, and potassium fertilizer treatment on the rhizosphere soil microbial community. A total of 30 strawberry rhizosphere soil samples (three from each treatment group) were collected and microbial community diversity was studied based on high-throughput sequencing of the 16S rRNA and ITS genes. After performing quality control on the 16S rRNA sequencing data, a total of 409,413 high-quality reads were obtained. On average, each sample yielded 13,381 reads, with a minimum of 11,771 (S1 Table). Similarly, after quality control of the ITS sequencing data, there were a total of 393,802 high-quality sequences (average = 13,126 and minimum = 11,245) (S2 Table).

**Fig 4:**
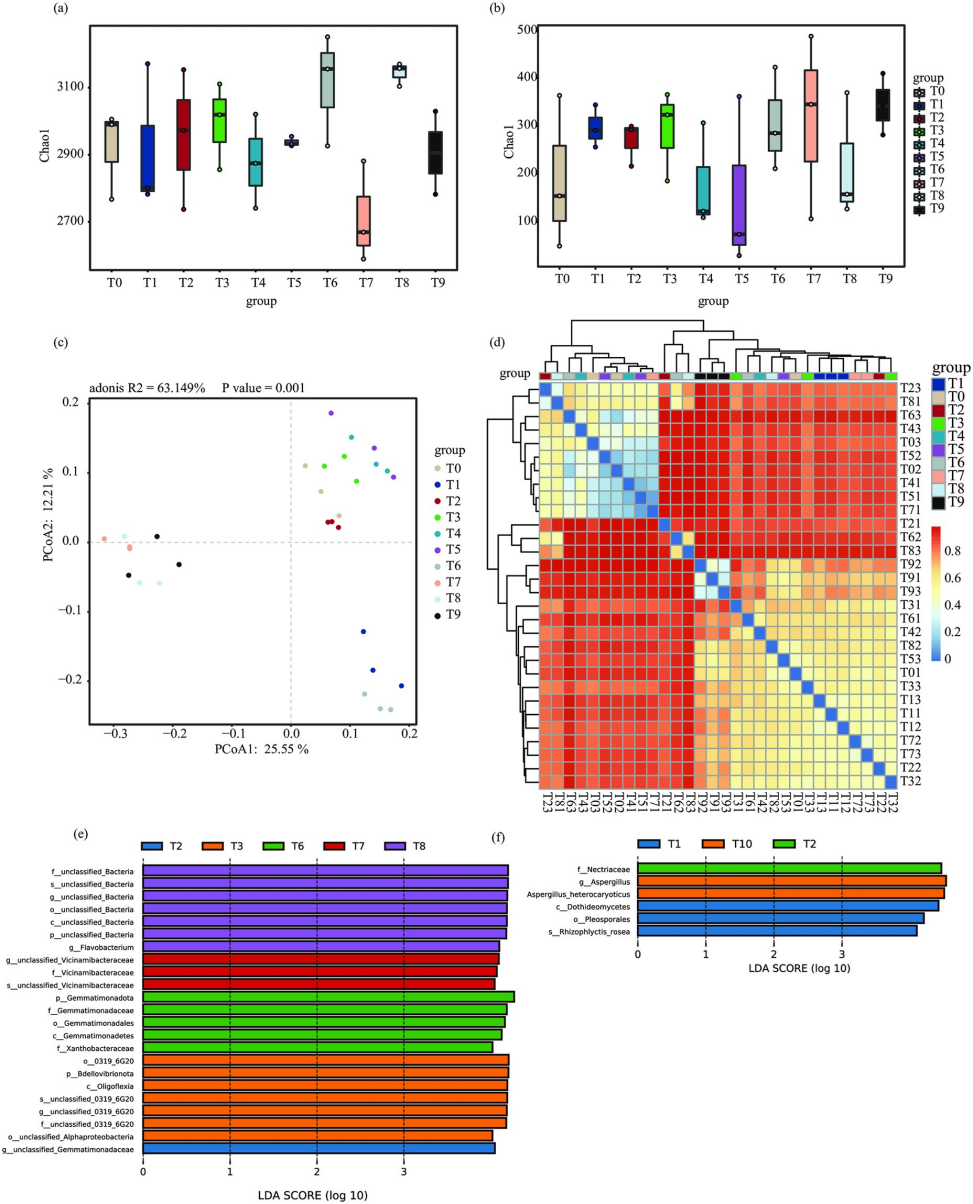
**(a, b)** Chao1 index showing bacterial **(a)** and fungal **(b)** community structure in the rhizosphere soil of strawberry plants treated with different levels of fertilizer. The horizontal bars within boxes represent medians. **(c)** Principal coordinate analysis (PCoA) based on the weighted Bray-Curtis distance of the bacterial communities. Permutational multivariate analysis of variance (PERMANOVA) was used to detect statistically significant differences between groups. **(d)** Heatmap based on the weighted Bray-Curtis distance of the fungal communities. **(e)** Significant differences in bacterial taxa between the fertilizer treatment groups as identified with linear discriminant analysis (LDA) coupled with effect size (LEfSe) analysis (LDA > 4 and *p* < 0.05). **(f)** Significant differences in fungal taxa between the fertilizer treatment groups as identified with LEfse analysis (LDA > 4 and *p* < 0.05).

For the subsequent taxonomic analysis, we focused on bacterial and fungal community composition at the phylum and genus levels. The groups with fertilizer applied were richest in the bacterial phylum Proteobacteria, followed by the phyla Bacteroidota, Acidobacteriota, Verrucomicrobiota, Patescibacteria, and Planctomycetota (S1a Fig). The most abundant fungal phylum was Monoblepharomycota, followed by Glomeromycota, Mucoromycota, and Zoopagomycota (S1b Fig). The T6 treatment group had the highest alpha diversity in both bacteria and fungi (Chao1 index). Interestingly, the T7 group had lower bacterial diversity than the T0 group (Fig 4a), but increased fungal diversity (Fig 4b). PCoA of the bacterial communities in the 10 groups showed that T0 clustered with the T2, T3, T4, and T5 groups *(p* > 0.05, Adonis), which were distinct from the T6 group (Fig 4c). In general, the combined application of N, P, and K fertilizer had minimal impacts on the fungal community structure (Fig 4d).

To identify differentially abundant microbial taxa between the treatment groups, we employed LEfSe. This analysis revealed 23 related bacterial taxa in five groups and six related fungal taxa in three groups. Specifically, p_Gemmatimonadota, f_Gemmatimonadaceae, o_Gemmatimonadales, c_Gemmatimonadetes, f_Xanthobacteraceae, g_Aspergillus, and s_Aspergillus_heterocaryoticus were significantly more abundant in the T6 group than in any other (Fig 4e). Conversely, in group T0 (Fig 4f), the abundance of these taxa was notably different.

### Relationships between soil composition and soil microorganisms

**Fig 5:**
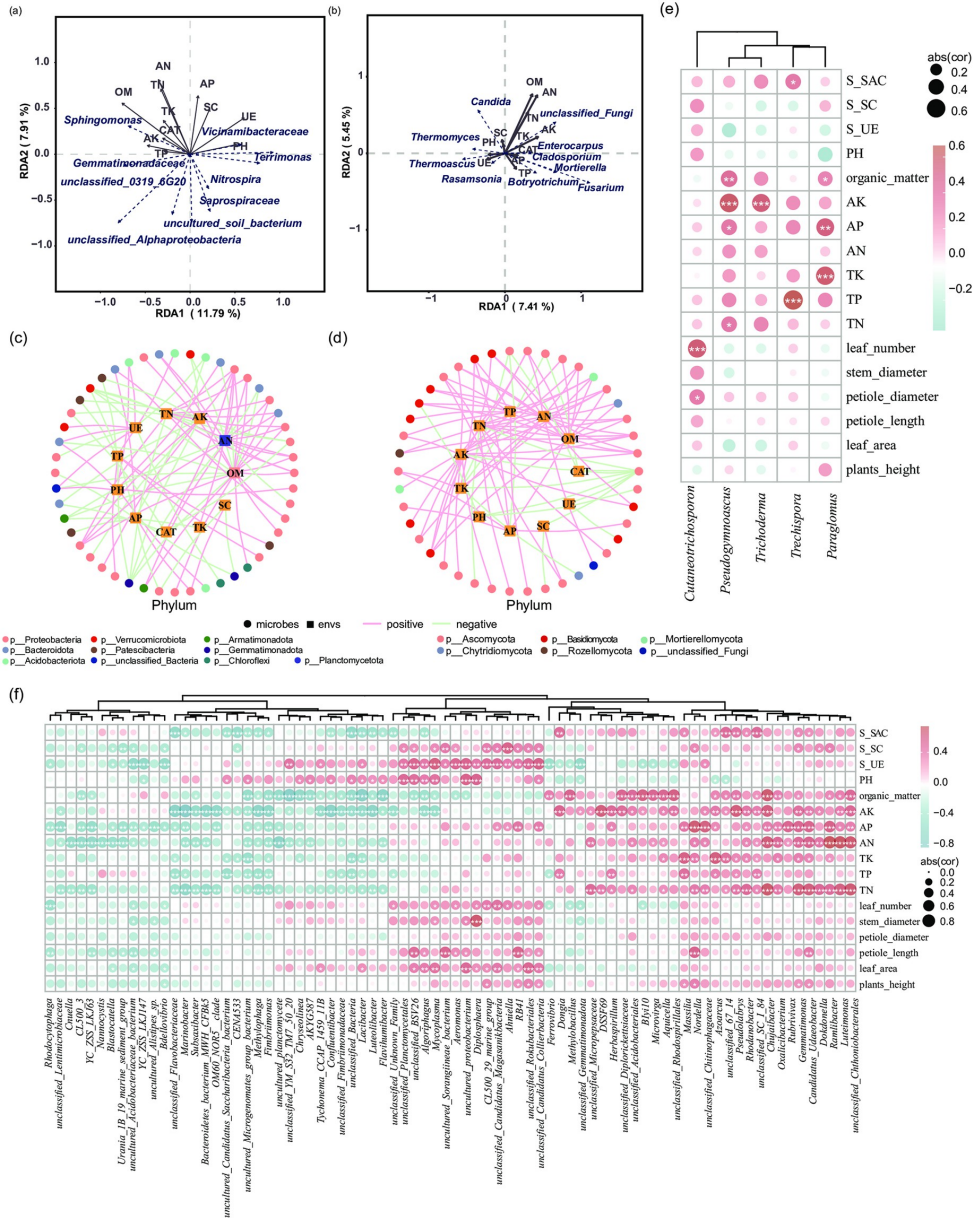
**(a, b)** Redundancy analysis of the impacts of soil composition on rhizosphere bacterial **(a)** and fungal **(b)** community structure. The arrow length represents the intensity of the indicated environmental factor’s influence on community changes (i.e., a long arrow represents a strong impact of the indicated environmental factor). The angle between the arrow and the axis represents the correlation between the indicated environmental factor and the axis; smaller angles correspond to higher correlations. The distance between each sample point and arrow indicates the strength of the effect of that environmental factor on the sample. The location of a sample in the same direction as an arrow indicates a positive correlation between the environmental factor and changes in the microbial community; the location of a sample in the opposite direction compared to an arrow indicates a negative correlation. **(c, d)** Correlation network analyses of soil microorganisms and soil physicochemical properties. Network analyses are shown for soil bacterial **(c)** and fungal **(d)** taxa and soil constituents. Circular nodes represent species (outer ring); square nodes represent environmental factors (inner ring). Circular node colors represent taxa at the genus level. Pink and light green lines indicate positive and negative correlations, respectively. **(e, f)** Spearman’s correlation coefficient values show the relationship between the presence of a fungal **(e)** or bacterial **(f)** taxon and the 17 phenotypic indices measured. Red and blue indicate positive and negative associations, respectively, between each measurement and the indicated species. *p < 0.05, **p < 0.01, ***p < 0.001.

Soil microbial community structure and composition are closely related to soil physical and chemical properties. Fertilization affected the bacterial and fungal community structures primarily by altering the soil carbon composition and extracellular enzyme activity. Redundancy analysis (RDA) showed that levels of soil organic matter, total nitrogen content, total phosphorus content, urease activity, and sucrase activity all affected the bacterial community (Fig 5a). Furthermore, the fungal community structure was affected by levels of soil organic matter, soil total nitrogen content, and soil total potassium content (Fig 5b). The key bacterial taxa that responded to environmental factors were p_Proteobacteria, p_Bacteroidota, and p_Acidobacteriota (Fig 5c), whereas the most responsive fungal taxa were p_Ascomycota, p_ Basidiomycota, and p_Chytridiomycota (Fig 5d). In addition, the relative abundances of the bacteria *Gemmatimonas* and *Udaeobacter* were positively correlated with soil N, P and K levels, and *Gemmatimonas* abundance was positively correlated with strawberry plant height (Fig 5e). Analysis of differences in the abundance of bacterial communities between treatment groups showed a significant increase in the number of *Gemmatimonas* in group T6 compared with group T0 (S2a Fig). Furthermore, the relative abundance of *Udaeobacter* was positively correlated with strawberry leaf length, whereas the relative abundances of *Methylophaga* and *Fimbriimonas* were negatively correlated with soil N, P, and K. The relative abundance of *Pseudogymnoascus* was positively correlated with soil P and K, and the relative abundance of *Trichoderma* was positively associated with soil K levels (Fig 5e).

## Discussion

Parameters such as plant height, leaf area, petiole diameter, petiole length, and stem diameter serve as vital indicators that provide insights into the intricate processes of plant growth. Understanding and monitoring these morphological characteristics hold paramount importance in evaluating the robustness of crop development [34]. The prudent and precise utilization of nitrogen, phosphorus, and potassium fertilizers assumes a critical role in facilitating the vigorous growth and optimal development of crops [35]. To investigate the ideal nitrogen, phosphorus, and potassium fertilizer application scheme for enhancing strawberry production, we conducted a comprehensive analysis of their effects on strawberry agronomic traits and soil characteristics during the 30–120 days period. Our study aimed to unravel the intricate relationship between these essential nutrients and their impact on both the growth parameters of strawberries and the underlying soil properties.

Soil enzymes are bioactive proteins predominantly synthesized by bacteria [36]. These enzymes are essential for the nutrient cycling process in soil, exerting a significant impact on soil fertility and ecological sustainability. Increased enzyme activity can enhance the capacity of nutrient uptake from plant-derived soils [37]. Studies have shown that different conditioning agents can improve soil environment and enhance soil enzyme activity [38]. Application of inorganic phosphorus fertilizer significantly enhances the activity of soil urease and catalase [39], while urea nitrogen application has a minor impact on soil enzyme activity [40]. We observed a declining trend in urease activity in the rhizosphere soil of strawberries, with significantly higher urease activity in the fertilizer application group compared to the non-fertilization treatment group. The nitrogen cycle in soil is an indicator of soil health and vitality [41]. Furthermore, the catalase activity in the rhizosphere soil exhibited an increasing trend during each period, and there was a correlation between urease activity, phosphorus (P), and soil catalase [42]. Additionally, alterations in the rhizosphere microorganism structure can impact the activities of numerous enzymes in the soil and indirectly influence soil nutrient levels.

The integrity, functioning, and long-term sustainability of soil ecosystems heavily rely on soil microbial diversity [43,44]. Numerous studies have consistently demonstrated that increased biodiversity contributes to the improvement of ecosystem quality and enhances the stability of microbial functions [45]. Rhizosphere bacterial strains have beneficial effects on strawberry plants under water stress [46]. Generally, the excessive application of nitrogen fertilizer resulted in a reduction in microbial diversity in both bulk and rhizosphere soil [47]. In our study, T7 and T9 demonstrated that excessive nitrogen application resulted in a reduction in bacterial species richness, while T8 and T9 exhibited a decrease in fungal species richness. These observations could be attributed to the selective pressure exerted by high concentrated AN and changes in soil pH on microbes [48]. Furthermore, the N level has the potential to regulate certain plant physiological characteristics, including leaf area index and chlorophyll content. These changes can alter the composition of root exudates or plant signaling, subsequently influencing the microbial community in the rhizosphere [49].

Different rates of nitrogen application have been observed to cause alterations in both soil bacterial diversity and community structure [50]. Soil bacterial diversity and community structure are influenced by varying nitrogen application rates. Our study corroborated this finding, as we observed the highest bacterial diversity in T6 and the highest fungal diversity in T7. PCoA analysis of bacterial communities and hierarchical cluster analysis of similarity indicated that each fertilizer application group formed a unique microbial community structure. In all treatments, the dominant phyla observed were Proteobacteria, Bacteroidetes, and Acidobacteria, which align with previous findings in strawberry soils [51]. These results suggest that excessive nitrogen levels may lead to a reduction in microbial community richness. However, this issue can be mitigated by utilizing different fertilizer ratios.

Rhizosphere bacteria fulfill crucial functions in plant nutrition and facilitate the growth of their host plants [52]. Strawberry plants possess the capacity to enlist microbes from the microbial reservoirs present in the soil into their rhizosphere, consequently augmenting the relative abundance of advantageous microorganisms engaged in nutrient assimilation and defense against soil-borne ailments [53]. The correlation analysis revealed a close relationship between strawberry agronomic traits and rhizosphere soil composition. We observed a positive correlation between *Gemmatimonas* and strawberry plant height, as well as a positive correlation with soil N, P, and K. Furthermore, the relative abundance of *Gemmatimonas* was significantly higher in the T6 treatment group. *Gemmatimonadetes*, widely distributed in the environment, possesses phylogenetic classification based on genes related to bacterial chlorophyll biosynthesis and their gene clusters associated with photosynthesis (PGC) [54]. However, there is limited research on the physiological and ecological aspects associated with *Gemmatimonadetes*. In subsequent studies, we can explore whether *Gemmatimonadetes* has antagonistic effects on harmful pathogenic bacteria in the soil [55]. Our results demonstrate that optimal application of nitrogen, phosphorus, and potassium can lead to cost reduction and improvement in microbial community structure, ultimately enhancing the phenotypic traits of strawberries.

## Conclusion

This study brings to light the effects of soil fertilization with inorganic NPK fertilizer on the strawberry growth and microbial communities and, in agreement with previous studies, revealed that different levels of inorganic fertilizer treatments affect drastically the microbial community structure and abundance in the agricultural soil. Higher, moderate, and lower doses of P, N, K fertilizer combination (T6) application boost the richness, abundance, and diversity of the bacteria, thereby enhancing the attainment of a stable community at the rhizosphere. Meanwhile, T7 fertilizer (Higher, lower, and higher doses of N, P, K respectively) combination showed the highest diversity of fungi. All groups (T1 to T9) with inorganic NPK fertilizer improved the growth of strawberry. These results showed that NPK fertilizer have very significant effects on the selection and enrichment of soil microbes’ community and agronomic traits of strawberry. Therefore, to achieve maximum results in promoting strawberry health, productivity, and the microbial community, an integrated fertilizer approach of using 225 kg/hm^2^ N, 180 kg/hm^2^ P, and 260 kg/hm^2^ K fertilizer is recommended.

## Supporting information

S1 Fig **S1-S2: (1)** Microbial composition is presented at phylum and genus levels; **(S2)**: Significant difference analysis was conducted on the bacterial.
(PDF)

S1 File Bacteria sample information. (XLSX)

S2 File Fungus sample information. (XLSX)

S3 File Phenotypic correlation data. (XLSX)

## References

[1] Zhang J, Balkovič J, Azevedo LB, Skalský R, Bouwman AF, Xu G, et al. Analyzing and modelling the effect of long-term fertilizer management on crop yield and soil organic carbon in China. Sci Total Environ. 2018;627:361–72. Epub 2018/02/11. doi: 10.1016/j.scitotenv.2018.01.090 . 29426159

[2] Zhang X, Xu M, Sun N, Xiong W, Huang S, Wu L. Modelling and predicting crop yield, soil carbon and nitrogen stocks under climate change scenarios with fertiliser management in the North China Plain. GEODERMA. 2016;265:176–86. doi: 10.1016/j.geoderma.2015.11.027

[3] Sauer T, Havlík P, Schneider UA, Schmid E, Kindermann G, Obersteiner M. Agriculture and resource availability in a changing world: The role of irrigation. WATER RESOURCES RESEARCH. 2010;46(6). doi: 10.1029/2009WR007729

[4] Lu Y, Song S, Wang R, Liu Z, Meng J, Sweetman AJ, et al. Impacts of soil and water pollution on food safety and health risks in China. Environ Int. 2015;77:5–15. Epub 2015/01/21. doi: 10.1016/j.envint.2014.12.010 . 25603422

[5] Zhou K, Sui Y-y, Xu X, Zhang J-y, Chen Y-m, Hou M, et al. The effects of biochar addition on phosphorus transfer and water utilization efficiency in a vegetable field in Northeast China. AGRICULTURAL WATER MANAGEMENT. 2018;210:324–9. doi: 10.1016/j.agwat.2018.08.007

[6] Kalt W, Forney CF, Martin A, Prior RL. Antioxidant capacity, vitamin C, phenolics, and anthocyanins after fresh storage of small fruits. J Agric Food Chem. 1999;47(11):4638–44. Epub 1999/12/20. doi: 10.1021/jf990266t . 10552863

[7] Garriga M, Retamales JB, Romero-Bravo S, Caligari PD, Lobos GA. Chlorophyll, anthocyanin, and gas exchange changes assessed by spectroradiometry in Fragaria chiloensis under salt stress. J Integr Plant Biol. 2014;56(5):505–15. Epub 2014/03/13. doi: 10.1111/jipb.12193 . 24618024

[8] Ruan J, Lee YH, Yeoung YR. Flowering and fruiting of day-neutral and ever-bearing strawberry cultivars in high-elevation for summer and autumn fruit production in Korea. Horticulture, Environment, and Biotechnology. 2013;54(2):109–20. doi: 10.1007/s13580-013-0185-9

[9] Rowley D, Black BL, Drost D, Feuz D. Late-season Strawberry Production Using Day-neutral Cultivars in High-elevation High Tunnels. HortScience horts. 2011;46(11):1480–5. doi: 10.21273/HORTSCI.46.11.1480

[10] Li H, Lascano RJ. Deficit irrigation for enhancing sustainable water use: Comparison of cotton nitrogen uptake and prediction of lint yield in a multivariate autoregressive state-space model. ENVIRONMENTAL AND EXPERIMENTAL BOTANY. 2011;71(2):224–31. doi: 10.1016/j.envexpbot.2010.12.007

[11] Dordas CA, Sioulas C. Safflower yield, chlorophyll content, photosynthesis, and water use efficiency response to nitrogen fertilization under rainfed conditions. INDUSTRIAL CROPS AND PRODUCTS. 2008;27(1):75–85. doi: 10.1016/j.indcrop.2007.07.020

[12] Deng X, Woodward FI. The Growth and Yield Responses of Fragaria ananassa to Elevated CO 2 and N Supply. ANNALS OF BOTANY. 1998;81(1):67–71. doi: 10.1006/anbo.1997.0535

[13] Tsialtas JT, Maslaris N. Effect of N Fertilization Rate on Sugar Yield and Non-Sugar Impurities of Sugar Beets (Beta vulgaris) Grown Under Mediterranean Conditions. JOURNAL OF AGRONOMY AND CROP SCIENCE. 2005;191(5):330–9. doi: 10.1111/j.1439-037X.2005.00161.x

[14] Li H, Li T, Fu G, Hu K. How strawberry plants cope with limited phosphorus supply: Nursery-crop formation and phosphorus and nitrogen uptake dynamics. JOURNAL OF PLANT NUTRITION AND SOIL SCIENCE. 2014;177(2):260–70. doi: 10.1002/jpln.201200654

[15] Ahmad M, Ishaq M, Shah WA, Adnan M, Fahad S, Saleem MH, et al. Managing Phosphorus Availability from Organic and Inorganic Sources for Optimum Wheat Production in Calcareous Soils. Sustainability [Internet]. 2022; 14(13).

[16] Solangi F, Zhu X, Khan S, Rais N, Majeed A, Sabir MA, et al. The Global Dilemma of Soil Legacy Phosphorus and Its Improvement Strategies under Recent Changes in Agro-Ecosystem Sustainability. ACS Omega. 2023;8(26):23271–82. doi: 10.1021/acsomega.3c00823 37426212 PMC10324088

[17] Grant CA, Flaten DN, Tomasiewicz DJ, Sheppard SC. The importance of early season phosphorus nutrition. CANADIAN JOURNAL OF PLANT SCIENCE. 2001;81(2):211–24. doi: 10.4141/P00-093

[18] Oosterhuis DM, Loka DA, Kawakami EM, Pettigrew WT. The physiology of potassium in crop production. Advances in Agronomy. 2014;126:203–33.

[19] Pettigrew WT. Potassium influences on yield and quality production for maize, wheat, soybean and cotton. Physiol Plant. 2008;133(4):670–81. Epub 2008/03/12. doi: 10.1111/j.1399-3054.2008.01073.x . 18331406

[20] Tsialtas IT, Shabala S, Baxevanos D, Matsi T. Effect of potassium fertilization on leaf physiology, fiber yield and quality in cotton (Gossypium hirsutum L.) under irrigated Mediterranean conditions. FIELD CROPS RESEARCH. 2016;193:94–103. doi: 10.1016/j.fcr.2016.03.010

[21] Xu L, Huang H-R, Yang L-T, Li Y-R. Combined application of NPK on yield quality of sugarcane applied through SSDI. Sugar Tech. 2010;12(2):104–7. doi: 10.1007/s12355-010-0021-9

[22] Ayoola O, Adeniyan O. Influence of poultry manure and NPK fertilizer on yield and yield components of crops under different cropping systems in south west Nigeria. African journal of biotechnology. 2006;5(15).

[23] Muhammad H, Fahad S, Saud S, Hassan S, Nasim W, Ali B, et al. A Paradigm Shift towards Beneficial Microbes Enhancing the Efficiency of Organic and Inorganic Nitrogen Sources for a Sustainable Environment. Land [Internet]. 2023; 12(3).

[24] Sener S, Fethiye N. Effects of Genotype and Fertilization on Fruit Quality in Several Harvesting Periods of Organic Strawberry Plantation. 2023:2319–1473.

[25] Pokhrel B, Laursen KH, Petersen KK. Yield, Quality, and Nutrient Concentrations of Strawberry (Fragaria ×ananassa Duch. cv. ’Sonata’) Grown with Different Organic Fertilizer Strategies. J Agric Food Chem. 2015;63(23):5578–86. Epub 2015/05/27. doi: 10.1021/acs.jafc.5b01366 . 26006727

[26] Sønsteby A, Opstad N, Myrheim U, Heide OM. Interaction of short day and timing of nitrogen fertilization on growth and flowering of ‘Korona’ strawberry (Fragaria×ananassa Duch.). SCIENTIA HORTICULTURAE. 2009;123(2):204–9. doi: 10.1016/j.scienta.2009.08.009

[27] Palaniswamy KM, Gomez KA. Length-Width Method for Estimating Leaf Area of Rice1. AGRONOMY JOURNAL. 1974;66(3):430–3. doi: 10.2134/agronj1974.00021962006600030027x

[28] Chaín JM, Tubert E, Graciano C, Castagno LN, Recchi M, Pieckenstain FL, et al. Growth promotion and protection from drought in Eucalyptus grandis seedlings inoculated with beneficial bacteria embedded in a superabsorbent polymer. Sci Rep. 2020;10(1):18221. Epub 2020/10/28. doi: 10.1038/s41598-020-75212-4 . 33106567 PMC7588442

[29] Callahan BJ, Wong J, Heiner C, Oh S, Theriot CM, Gulati AS, et al. High-throughput amplicon sequencing of the full-length 16S rRNA gene with single-nucleotide resolution. Nucleic Acids Res. 2019;47(18):e103. Epub 2019/07/04. doi: 10.1093/nar/gkz569 . 31269198 PMC6765137

[30] Gardes M, Bruns TD. ITS primers with enhanced specificity for basidiomycetes—application to the identification of mycorrhizae and rusts. Mol Ecol. 1993;2(2):113–8. Epub 1993/04/01. doi: 10.1111/j.1365-294x.1993.tb00005.x . 8180733

[31] Bolyen E, Rideout JR, Dillon MR, Bokulich NA, Abnet CC, Al-Ghalith GA, et al. Reproducible, interactive, scalable and extensible microbiome data science using QIIME 2. NATURE BIOTECHNOLOGY. 2019;37(8):852–7. doi: 10.1038/s41587-019-0209-9 31341288 PMC7015180

[32] Quast C, Pruesse E, Yilmaz P, Gerken J, Schweer T, Yarza P, et al. The SILVA ribosomal RNA gene database project: improved data processing and web-based tools. NUCLEIC ACIDS RESEARCH. 2013;41(D1):D590–D6. doi: 10.1093/nar/gks1219 23193283 PMC3531112

[33] Segata N, Izard J, Waldron L, Gevers D, Miropolsky L, Garrett WS, et al. Metagenomic biomarker discovery and explanation. GENOME BIOLOGY. 2011;12(6):R60. doi: 10.1186/gb-2011-12-6-r60 21702898 PMC3218848

[34] Arend D, Lange M, Pape JM, Weigelt-Fischer K, Arana-Ceballos F, Mücke I, et al. Quantitative monitoring of Arabidopsis thaliana growth and development using high-throughput plant phenotyping. Sci Data. 2016;3:160055. Epub 2016/08/17. doi: 10.1038/sdata.2016.55 . 27529152 PMC4986541

[35] Zhang H, Zeng Z, Zou Z, Zeng F. Climate, Life Form and Family Jointly Control Variation of Leaf Traits. Plants (Basel). 2019;8(8). Epub 2019/08/17. doi: 10.3390/plants8080286 . 31416214 PMC6724092

[36] Xiao XY, Wang MW, Zhu HW, Guo ZH, Han XQ, Zeng P. Response of soil microbial activities and microbial community structure to vanadium stress. Ecotoxicol Environ Saf. 2017;142:200–6. Epub 2017/04/16. doi: 10.1016/j.ecoenv.2017.03.047 . 28411515

[37] Zhaolei L, Naishun B, Jun C, Xueping C, Manqiu X, Feng W, et al. Effects of long-term cultivation of transgenic Bt rice (Kefeng-6) on soil microbial functioning and C cycling. Sci Rep. 2017;7(1):4647. Epub 2017/07/07. doi: 10.1038/s41598-017-04997-8 . 28680066 PMC5498577

[38] Liu B, Xia H, Jiang C, Riaz M, Yang L, Chen Y, et al. 14 year applications of chemical fertilizers and crop straw effects on soil labile organic carbon fractions, enzyme activities and microbial community in rice-wheat rotation of middle China. SCIENCE OF THE TOTAL ENVIRONMENT. 2022;841:156608. doi: 10.1016/j.scitotenv.2022.156608 35700778

[39] Wu W, Wu J, Liu X, Chen X, Wu Y, Yu S. Inorganic phosphorus fertilizer ameliorates maize growth by reducing metal uptake, improving soil enzyme activity and microbial community structure. Ecotoxicol Environ Saf. 2017;143:322–9. Epub 2017/06/05. doi: 10.1016/j.ecoenv.2017.05.039 . 28578263

[40] Davies B, Coulter JA, Pagliari PH. Soil Enzyme Activity Behavior after Urea Nitrogen Application. Plants (Basel). 2022;11(17). Epub 2022/09/10. doi: 10.3390/plants11172247 . 36079628 PMC9460541

[41] Adetunji AT, Lewu FB, Mulidzi R, Ncube B. The biological activities of β-glucosidase, phosphatase and urease as soil quality indicators: a review. Journal of Soil Science and Plant Nutrition. 2017;17(3):794–807.

[42] Khan A, Jiang H, Bu J, Adnan M, Gillani SW, Zhang M. An insight to rhizosphere bacterial community composition and structure of consecutive winter-initiated sugarcane ratoon crop in Southern China. BMC Plant Biol. 2022;22(1):74. Epub 2022/02/21. doi: 10.1186/s12870-022-03463-6 . 35183114 PMC8857817

[43] Kennedy A, Smith K. Soil microbial diversity and the sustainability of agricultural soils. PLANT AND SOIL. 1995;170:75–86.

[44] Liu SY, Wei CY, Tong Y, Chen W, Han ZY, Zeng DQ, et al. Cyperus rotundus L. drives arable soil infertile by changing the structure of soil bacteria in the rhizosphere, using a maize field as an example. Environ Sci Pollut Res Int. 2022;29(52):79579–93. Epub 2022/06/18. doi: 10.1007/s11356-022-21480-8 . 35715673

[45] Chaer G, Fernandes M, Myrold D, Bottomley P. Comparative Resistance and Resilience of Soil Microbial Communities and Enzyme Activities in Adjacent Native Forest and Agricultural Soils. MICROBIAL ECOLOGY. 2009;58(2):414–24. doi: 10.1007/s00248-009-9508-x 19330551

[46] Paliwoda D, Mikiciuk G, Mikiciuk M, Kisiel A, Sas-Paszt L, Miller T. Effects of Rhizosphere Bacteria on Strawberry Plants (Fragaria × ananassa Duch.) under Water Deficit. Int J Mol Sci. 2022;23(18). Epub 2022/09/24. doi: 10.3390/ijms231810449 . 36142361 PMC9499335

[47] Wang Q, Ma M, Jiang X, Guan D, Wei D, Zhao B, et al. Impact of 36 years of nitrogen fertilization on microbial community composition and soil carbon cycling-related enzyme activities in rhizospheres and bulk soils in northeast China. APPLIED SOIL ECOLOGY. 2019;136:148–57.

[48] Li Y-R, Song X-P, Wu J-M, Li C-N, Liang Q, Liu X-H, et al. Sugar industry and improved sugarcane farming technologies in China. Sugar Tech. 2016;18:603–11.

[49] Basal O, Szabó A. The effects of drought and nitrogen on soybean (Glycine max (L.) Merrill) physiology and yield. International Journal of Agricultural and Biosystems Engineering. 2018;12(9):260–5.

[50] Zhang M, Wang W, Zhang Y, Teng Y, Xu Z. Effects of fungicide iprodione and nitrification inhibitor 3, 4-dimethylpyrazole phosphate on soil enzyme and bacterial properties. Sci Total Environ. 2017;599–600:254–63. Epub 2017/05/10. doi: 10.1016/j.scitotenv.2017.05.011 . 28477482

[51] Huang Y, Xiao X, Huang H, Jing J, Zhao H, Wang L, et al. Contrasting beneficial and pathogenic microbial communities across consecutive cropping fields of greenhouse strawberry. APPLIED MICROBIOLOGY AND BIOTECHNOLOGY. 2018;102(13):5717–29. doi: 10.1007/s00253-018-9013-6 29704041

[52] Nam JH, Thibodeau A, Qian YL, Qian MC, Park SH. Multidisciplinary evaluation of plant growth promoting rhizobacteria on soil microbiome and strawberry quality. AMB Express. 2023;13(1):18. Epub 2023/02/17. doi: 10.1186/s13568-023-01524-z . 36795258 PMC9935790

[53] Lazcano C, Boyd E, Holmes G, Hewavitharana S, Pasulka A, Ivors K. The rhizosphere microbiome plays a role in the resistance to soil-borne pathogens and nutrient uptake of strawberry cultivars under field conditions. Sci Rep. 2021;11(1):3188. Epub 2021/02/06. doi: 10.1038/s41598-021-82768-2 . 33542451 PMC7862632

[54] Zeng Y, Nupur, Wu N, Madsen AM, Chen X, Gardiner AT, et al. Gemmatimonas groenlandica sp. nov. Is an Aerobic Anoxygenic Phototroph in the Phylum Gemmatimonadetes. Front Microbiol. 2020;11:606612. Epub 2021/02/02. doi: 10.3389/fmicb.2020.606612 . 33519753 PMC7844134

[55] Drobek M, Cybulska J, Gałązka A, Feledyn-Szewczyk B, Marzec-Grządziel A, Sas-Paszt L, et al. The Use of Interactions Between Microorganisms in Strawberry Cultivation (Fragaria x ananassa Duch.). Front Plant Sci. 2021;12:780099. Epub 2021/12/18. doi: 10.3389/fpls.2021.780099 . 34917112 PMC8668414

