# Supplementary material for: Effects of multiple N, P, and K fertilizer combinations on strawberry growth and the microbial community
Source: PLoS One. 2023 Nov 27;18(11):e0293088. doi: 10.1371/journal.pone.0293088 (PMC10681299; doi:10.1371/journal.pone.0293088)
Supplement: S1 Fig — S1-S2: (1) Microbial composition is presented at phylum and genus levels; (S2): Significant difference analysis was conducted on the bacterial. (PDF) [file pone.0293088.s001.pdf]

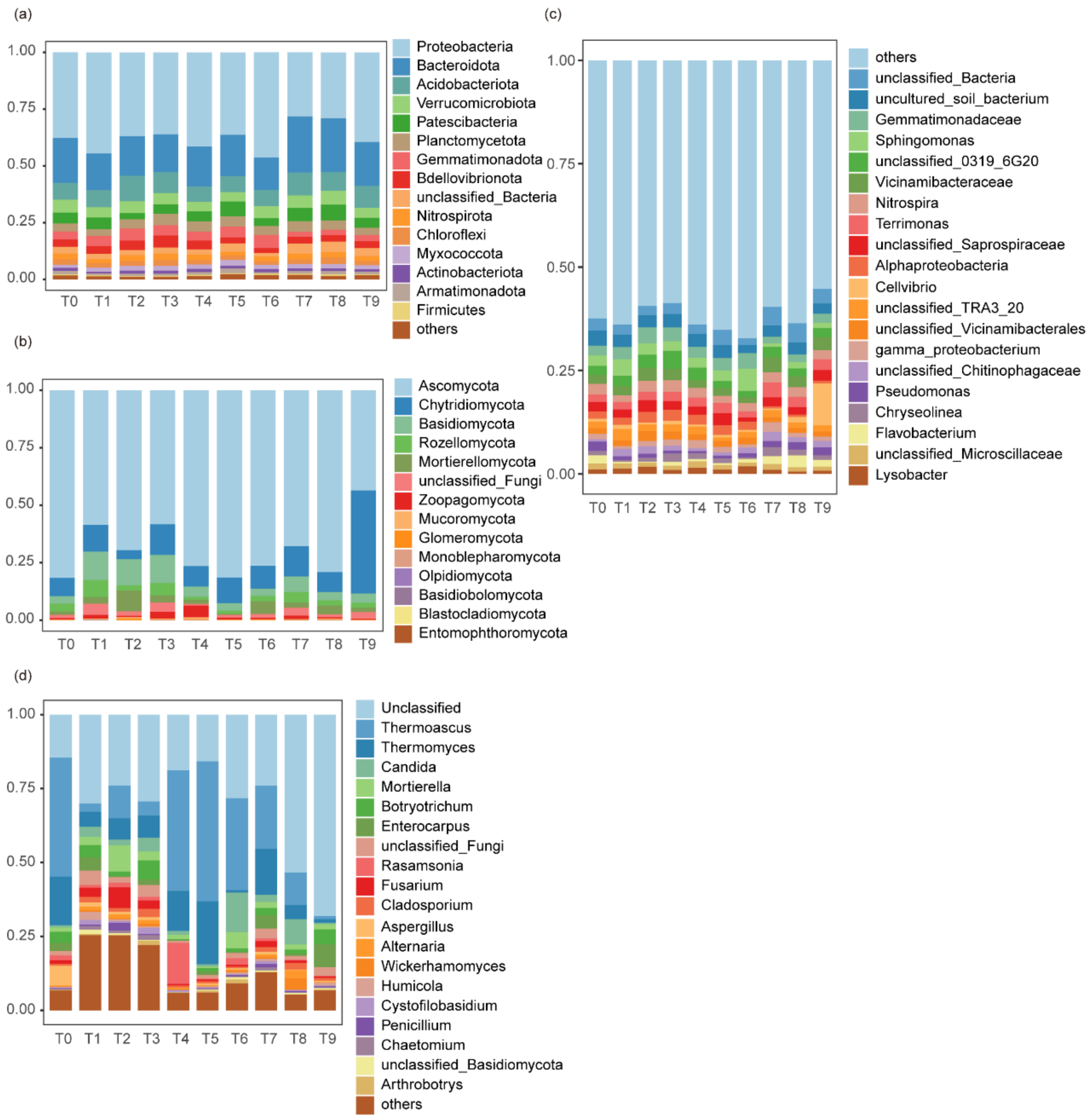

Figure S1. Microbial composition is presented at phylum and genus levels. Each bar graph represents the average relative abundance of each taxon within a fertilization group. (**a**) The top 15 bacterial phyla with the highest relative abundance in soil, while phyla with low relative abundance are classified as "others". (**b**) The top 15 fungal phyla with the highest relative abundance in soil, while phyla with low relative abundance are classified as "others". (**c**) The top 20 bacterial genera with the highest relative abundance in soil, while genera with low relative abundance are classified as "others". (**d**) The top 20 fungal genera with the highest relative abundance in soil, while genera with lower relative abundance are classified as "others".

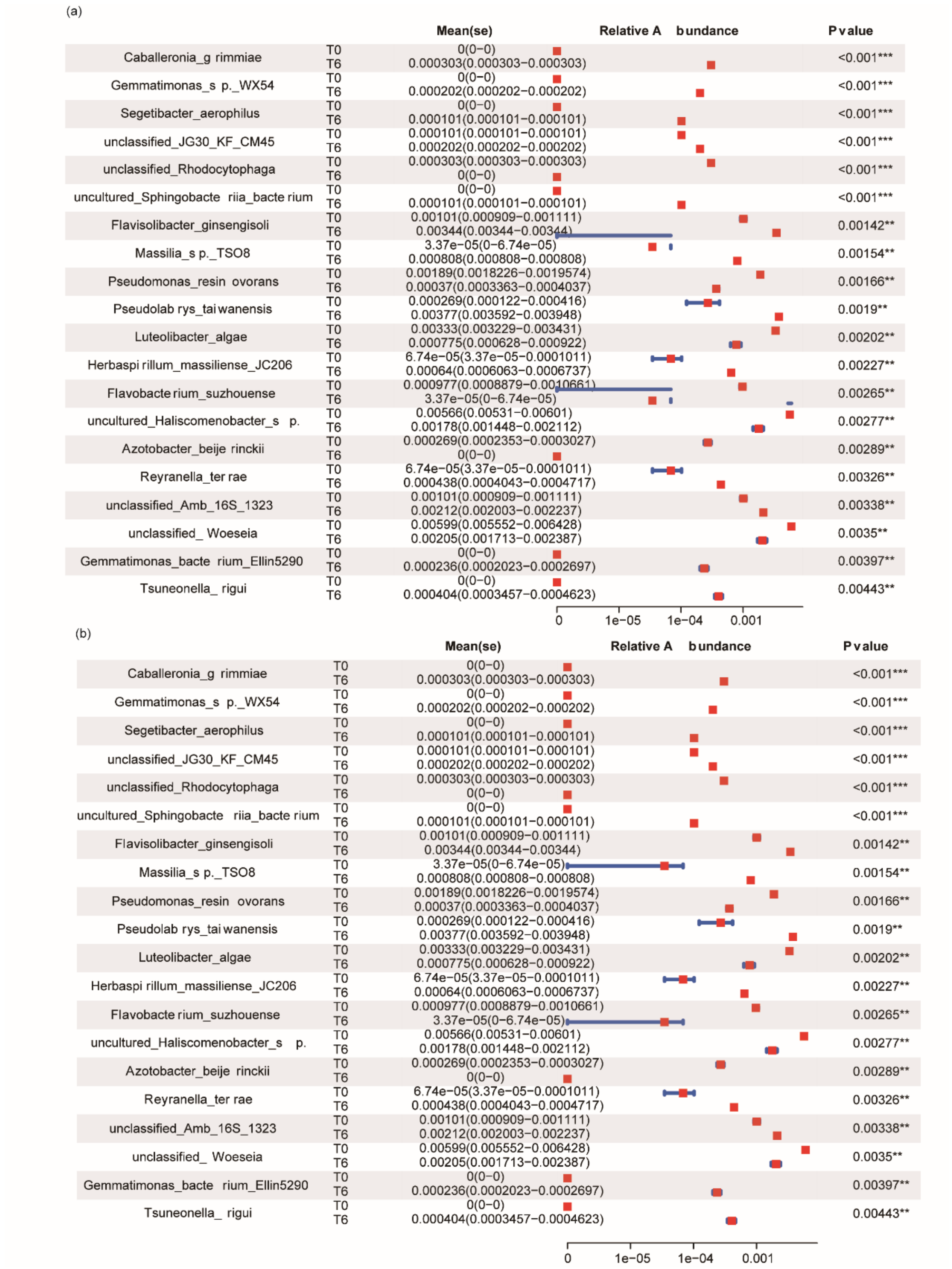

Figure S2. Significant difference analysis was conducted on the bacterial (**a**) species and fungal (**b**) genus levels for the T0 and T6 samples. The Metastats software was employed to perform a T-test on species abundance data between groups. The first column provides species classification information, the second column indicates the group, the third column displays the mean abundance and standard error, and the fourth column presents the relative abundance histogram for each group. The P-value represents the significance level of the hypothesis test.
